# Xpert MTB/RIF Ultra for the diagnosis of HIV-associated tuberculous meningitis: a prospective validation study

**DOI:** 10.1016/S1473-3099(19)30550-X

**Published:** 2020-03

**Authors:** Fiona V Cresswell, Lillian Tugume, Nathan C Bahr, Richard Kwizera, Ananta S Bangdiwala, Abdu K Musubire, Morris Rutakingirwa, Enock Kagimu, Edwin Nuwagira, Edward Mpoza, Joshua Rhein, Darlisha A Williams, Conrad Muzoora, Daniel Grint, Alison M Elliott, David B Meya, David R Boulware, ASTRO-CM team

**Affiliations:** a Clinical Research Department, London School of Hygiene & Tropical Medicine, London, UK; b Tropical Epidemiology Group, London School of Hygiene & Tropical Medicine, London, UK; c Infectious Diseases Institute, College of Health Sciences, Makerere University, Kampala, Uganda; d Medical Research Council/Uganda Virus Research Institute and London School of Hygiene & Tropical Medicine Uganda Research Unit, Entebbe, Uganda; e Division of Infectious Diseases, Department of Medicine, University of Kansas, Kansas City, KS, USA; f Division of Infectious Diseases and International Medicine, Department of Medicine, University of Minnesota, Minneapolis, MN, USA; g Division of Biostatistics, School of Public Health, University of Minnesota, Minneapolis, MN, USA; h Mbarara University of Science and Technology, Mbarara, Uganda

## Abstract

**Introduction:**

Tuberculous meningitis accounts for 1–5% of tuberculosis cases. Diagnostic delay contributes to poor outcomes. We evaluated the performance of the new Xpert MTB/RIF Ultra (Xpert Ultra) for tuberculous meningitis diagnosis.

**Methods:**

In this prospective validation study, we tested the cerebrospinal fluid (CSF) of adults presenting with suspected meningitis (ie, headache or altered mental status with clinical signs of meningism) to the Mulago National Referral Hospital and Mbarara Regional Referral Hospital in Uganda. We centrifuged the CSF, resuspended the cell pellet in 2 mL CSF, and tested 0·5 mL aliquots with Xpert Ultra, Xpert MTB/RIF (Xpert), and mycobacterial growth indicator tube (MGIT) culture. We quantified diagnostic performance against the uniform case definition of probable or definite tuberculous meningitis and a composite microbiological reference standard.

**Findings:**

From Nov 25, 2016, to Jan 24, 2019, we screened 466 adults with suspected meningitis and tested 204 for tuberculous meningitis. Uniform clinical case definition classified 51 participants as having probable or definite tuberculous meningitis. Against this uniform case definition, Xpert Ultra had 76·5% sensitivity (95% CI 62·5–87·2; 39 of 51 patients) and a negative predictive value of 92·7% (87·6–96·2; 153 of 165), compared with 55·6% sensitivity (44·0–70·4; 25 of 45; p=0·0010) and a negative predictive value of 85·8% (78·9–91·1; 121 of 141) for Xpert and 61·4% sensitivity (45·5–75·6; 27 of 44; p=0·020) and negative predictive value of 85·2% (77·4–91·1; 98 of 115) for MGIT culture. Against the composite microbiological reference standard, Xpert Ultra had sensitivity of 92·9% (80·5–98·5; 39 of 42), higher than Xpert at 65·8% (48·6–80·4; 25 of 38; p=0·0063) and MGIT culture at 72·2% (55·9–86·2; 27 of 37; p=0·092). Xpert Ultra detected nine tuberculous meningitis cases missed by Xpert and MGIT culture.

**Interpretation:**

Xpert Ultra detected tuberculous meningitis with higher sensitivity than Xpert and MGIT culture in this HIV-positive population. However, with a negative predictive value of 93%, Xpert Ultra cannot be used as a rule-out test. Clinical judgment and novel highly sensitive point-of-care tests are still required.

**Funding:**

Wellcome Trust, National Institute of Health, National Institute of Neurologic Diseases and Stroke, Fogarty International Center, and National Institute of Allergy and Infectious Diseases.

## Introduction

Worldwide, *Mycobacterium tuberculosis* affected more than 10 million people in 2018, with devastating consequences, including around 1·5 million deaths.^1^ Tuberculous meningitis, the most serious form of tuberculosis infection, accounts for 1–5% of new cases of tuberculosis and results in death or substantial disability in more than half of those affected.^2^ Outcomes are particularly poor in patients with HIV co-infection, which can triple the risk of death from tuberculous meningitis, reaching approximately 50%.^3^ Another major driver of poor outcomes is diagnostic delay.^4^

Cerebrospinal fluid (CSF) smear microscopy with Ziehl–Neelsen staining for acid-fast bacilli is the cheapest and most widely available test for tuberculous meningitis diagnosis, but it is insensitive in most settings without expert microscopists.^5^, ^6^, ^7^ Culture takes a minimum of 2 weeks to provide results (too slow for clinical utility), has only moderate sensitivity (30–60%), and is not readily available in most settings within low-income tuberculosis-endemic countries.^8^, ^6^ Xpert MTB/RIF (Xpert; Cepheid, Sunnyvale, CA, USA)—a rapid, automated, cartridge-based molecular test—was endorsed by WHO in 2015 as the best initial test for tuberculous meningitis.^9^ Xpert has been deployed in 130 of 145 countries eligible for concessional pricing as of 2016.^10^ Xpert provides 45–67% sensitivity to detect microbiologically proven tuberculous meningitis, meaning a negative result does not provide adequate confidence that tuberculous meningitis is not present.^8^, ^11^ Thus, empirical antituberculous therapy for tuberculous meningitis, with its associated drug toxicities, drug–drug interactions, pill burden, and cost, is still commonly used, often unnecessarily.

**Research in context**

**Evidence before this study**

We searched PubMed Central for reports of Xpert MTB/RIF Ultra (Xpert Ultra) for the diagnosis of tuberculosis using the terms (“Xpert MTB/RIF Ultra” or “Xpert Ultra”) and (“tuberculosis” or “TB” or “tuberculous meningitis” or “TBM” or “extrapulmonary”). The search was done on Feb 14, 2019, with no search date or language restrictions.

In-vitro studies of Xpert MTB/RIF (Xpert) and Xpert Ultra assays have been done on sputum samples spiked with decreasing numbers of *Mycobacterium tuberculosis* H37Rv colony-forming units (CFUs), which found the limit of detection to be 16 CFUs per mL for Xpert Ultra versus 113 CFUs per mL for Xpert—a seven-times reduction in the limit of detection. This improvement signified a promising step for the diagnosis of paucibacillary tuberculosis. Since then, several prospective and retrospective studies have evaluated Xpert Ultra in diagnosis of adult pulmonary tuberculosis (four), paediatric tuberculosis (two), extrapulmonary tuberculosis (two), paucibacillary tuberculosis (one), and tuberculous meningitis (one), all of which have found Xpert Ultra to be more sensitive than Xpert. The largest prospective diagnostic accuracy study to date, including 1753 participants with suspected pulmonary tuberculosis, found Xpert Ultra to have superior sensitivity to Xpert in smear-negative pulmonary tuberculosis (63% *vs* 46%) and in HIV-associated pulmonary tuberculosis (90% *vs* 77%), albeit with a more modest difference of 5·4 percentage points in the general pulmonary tuberculosis population. To date, only Bahr and colleagues have specifically focused on tuberculous meningitis, testing 129 cryopreserved CSF samples from HIV-positive Ugandan adults and finding sensitivities of 70% (16 of 23 patients) for Xpert Ultra and 43% (ten of 23) for Xpert against a uniform reference standard of probable or definite tuberculous meningitis. The sensitivity against a composite microbiological reference standard was 95% (21 of 22) for Xpert Ultra and 45% (ten of 22) for Xpert (p=0·0010).

**Added value of this study**

To our knowledge, this prospective study is the largest to date to investigate the diagnostic performance of Xpert Ultra for tuberculous meningitis. It corroborates the findings from cryopreserved CSF samples and adds to the data on use of Xpert Ultra in HIV-positive populations and in paucibacillary forms of tuberculosis. We tested each sample with Xpert and Xpert Ultra to allow direct head-to-head comparisons of the performance of the two assays. To optimise the uniform case definition reference standard, we endeavoured to exclude other non-tuberculosis causes of meningitis by testing CSF with a meningoencephalitis PCR panel (testing for 14 viral, bacterial, and fungal pathogens) and offering post-mortem examinations when possible for participants who died without a microbiologically confirmed diagnosis.

**Implications of all the available evidence**

Our results support the WHO recommendations to use Xpert Ultra as the initial test for suspected tuberculous meningitis. With its run-time of 84 min, Xpert Ultra, in the context of appropriate infrastructure, holds potential to provide same-day results and facilitate prompt tuberculous meningitis treatment. Nonetheless, Xpert Ultra's imperfect negative predictive value (92·7% [95% CI 87·6–96·2] *vs* the uniform clinical case definition in this study) means that clinical judgment must override a negative Xpert Ultra result and empirical tuberculosis treatment is still warranted in cases where there is a high index of suspicion.

Subsequently, Xpert MTB/RIF Ultra (Xpert Ultra) was developed, which has a larger chamber (50 μL) for DNA amplification than Xpert, allowing twice the volume of sample to reach the PCR reaction. Xpert Ultra also has two new multicopy DNA targets (IS6110 and IS1081), incorporates fully nested nucleic acid amplification, and uses melting temperature-based analysis instead of real-time PCR to improve the accuracy of rifampicin-resistance detection. In vitro, these changes have made the limit of detection of *M tuberculosis* seven times lower (16 *vs* 113 colony forming units [CFUs] per mL).^12^, ^13^ Another important benefit of Xpert Ultra is the shortened run time of 84 min (compared with 112 min for Xpert), enabling an extra five tests to be run on each module in a working day, equating to an extra 40 tests per day on an eight-module unit. To upgrade from Xpert, only new software and the Xpert Ultra cartridges are required—the same instrument can be used. Previous studies have shown that Xpert Ultra's sensitivity is markedly improved over Xpert's in smear-negative pulmonary tuberculosis (63% *vs* 46%),^14^ HIV-associated pulmonary tuberculosis (90% *vs* 77%),^14^ and paediatric tuberculosis (74% *vs* 63%).^15^ The largest study to date of Xpert Ultra in tuberculous meningitis used cryopreserved CSF samples and found sensitivities of 95% (95% CI 77–99) for Xpert Ultra and the same sensitivity of 45% (24–68) for each of Xpert and mycobacterial growth indicator tube (MGIT) culture against a composite microbiological reference standard.^8^ Against the uniform clinical case definition of probable or definite tuberculous meningitis,^16^ sensitivity was 70% (47–87; 16 of 23 patients) for Xpert Ultra and 43% (23–66; ten of 23) for both culture and Xpert.^8^ Four additional small studies on CSF (containing a minimum of four and a maximum of 43 samples) corroborated the increased sensitivity of Xpert Ultra.^17^, ^18^, ^19^, ^20^ Additional larger studies using real-time CSF samples and prespecified reference standards are needed to better inform the current understanding of Xpert Ultra's performance in tuberculous meningitis.

The aim of this study was to prospectively assess the diagnostic accuracy of Xpert Ultra for tuberculous meningitis compared with Xpert using fresh CSF specimens from an HIV-positive population.

## Methods

### Study design and population

In this diagnostic accuracy study, we prospectively evaluated adults (aged ≥18 years) presenting consecutively to Mulago National Referral Hospital, Kampala, and Mbarara Regional Referral Hospital, Mbarara, Uganda, with suspected meningitis (headache for >3 days or altered mental status [Glasgow Coma Scale <15] with clinical signs of meningism at examination—ie, neck stiffness or Kernig's sign). The period of enrolment ran from Nov 26, 2016, to Jan 24, 2019, at Mulago National Referral Hospital and from Nov 25, 2016, to June 13, 2017, at Mbarara Regional Referral Hospital. Ethical approval was obtained from the Mulago Hospital, London School of Hygiene & Tropical Medicine, and University of Minnesota institutional review boards and Uganda National Council of Science and Technology, and informed consent was obtained from participants or their surrogate (in patients with altered mental status) as part of the screening process for a meningitis clinical trial (NCT01802385) that subsequently rolled into an observational diagnostic study. The study was conducted in line with the Standards for Reporting Diagnostic Accuracy Studies.^21^

### Procedures

Given the high prevalence of cryptococcus and the similar initial presentations between cryptococcal meningitis and tuberculous meningitis, fingerstick cryptococcal antigen lateral flow assay (LFA; Immy, Norman, OK, USA) was done at the participant's bedside. Thereafter, lumbar puncture was done for all participants, CSF opening pressure was recorded, and CSF was collected into a sterile tube with a target volume of more than 6 mL. At the bedside, CSF glucose and lactate were measured from a drop of CSF collected into an Eppendorf tube using a handheld OneTouch glucose meter (OneTouch; Lifescan, Inverness, UK) and a point-of-care lactate meter (Nova Biomedical; Waltham, MA, USA). Xpert and Xpert Ultra results were returned to the study team within 24 h to guide treatment decisions.

Clinical history, physical examination, and detailed neurological assessment findings were recorded as recommended by the Tuberculous Meningitis International Research Consortium.^22^ Other diagnostic tests, including urine tuberculosis lipoarabinomannan (Alere Determine; Alere, Waltham, MA, USA), chest radiograph, abdominal ultrasonography, brain imaging, and sputum Xpert, were done as clinically indicated and when locally available.

### Microbiological testing

Laboratory CSF testing included white blood cell count, lymphocyte percentage, differential Gram stain, aerobic bacterial culture, Ziehl–Neelsen acid-fast bacilli stain (Kampala only), total protein, CSF cryptococcal antigen, and Biofire FilmArray meningoencephalitis PCR panel (bioMérieux; Durham, NC, USA) for 14 meningoencephalitis pathogens (*Streptococcus pneumoniae, Neisseria meningitidis, Listeria monocytogenes, Haemophilus influenzae, Streptococcus agalactiae, Escherichia coli,* herpes simplex virus types 1 and 2, cytomegalovirus, varicella zoster virus, human herpes virus 6, enterovirus, human parechovirus, and cryptococcus). All samples that were negative for CSF cryptococcal antigen underwent comprehensive tuberculosis diagnostic studies. Samples positive for cryptococcal antigen underwent fungal culture and, in the event of fungal growth, quantitative fungal culture count.

For tuberculosis analysis, the CSF was centrifuged at 3000 g for 20 min to concentrate bacilli into the cell pellet. Supernatant was pipetted out to leave a residual volume of 2 mL in which the cell pellet was re-suspended by vortexing. The resuspended cell pellet was then divided into four 0·5 mL aliquots for Xpert, Xpert Ultra, MGIT culture, and cryopreservation at −80°C in case of invalid results from Xpert or Xpert Ultra or for future sequencing work. The aliquots for Xpert and Xpert Ultra were mixed with 1·5 mL of sample reagent, shaken vigorously, allowed to sit for 15 min, and then run on the Cepheid platform. The 0·5 mL aliquot for MGIT culture was added to the MGIT tube and incubated in a Bactec 960 instrument (Becton Dickinson; Franklin Lakes, NJ, USA). The algorithm for CSF diagnostic testing is illustrated in the appendix (p 2). Where the volume of CSF was below 6 mL, a stepwise approach was used to maximise the likelihood of yielding a diagnosis for the participant, as shown in the appendix (p 4). At the clinician's discretion, participants with a positive test for CSF cryptococcal antigen were allowed to undergo tuberculous meningitis testing as cryptococcosis coinfection with tuberculous meningitis does occur, although rarely.^23^ In Mbarara, tuberculosis testing was done at the Médecins Sans Frontières Epicentre laboratory. In Kampala, testing was done at the Makerere University Microbiology Laboratory, Makerere University Mycobacteriology (BSL-3) laboratory (MGIT culture), and the Infectious Diseases Institute Translational laboratory (Xpert and Xpert Ultra). Clinical information was not available to the performers of the index or reference tests. The same laboratory technologist performed Xpert and Xpert Ultra, but MGIT results were read and reported by a different technologist.

Xpert results are reported as follows: *M tuberculosis* not detected; invalid, if the internal quality control checks fail; or, if *M tuberculosis* DNA is detected, a semi-quantitative category of very low, low, medium, or high is given. Xpert Ultra results are reported in the same way but with the addition of the trace semi-quantitative category for the lowest bacillary loads (less than approximately 113 CFUs per mL). Rifampicin resistance is reported as indeterminate for tests positive in the trace category as the DNA quantity is too low to adequately detect rifampicin resistance conferring mutations.

For participants who died during hospitalisation at Mulago hospital and did not have a microbiologically confirmed diagnosis, the families were invited to provide informed consent for post-mortem examination in the Makerere University mortuary by a trained pathologist. Post-mortem examinations were unavailable in Mbarara. Additionally, CSF samples collected from participants with cryptococcal meningitis and who survived without tuberculosis therapy (ie, negative controls) were run on Xpert Ultra from the same parent study (NCT01802385).

### Statistical analysis

We assessed the diagnostic accuracy of Xpert Ultra against two reference standards. For the primary reference standard, we used the consensus uniform case definition of probable (≥10 points on the diagnostic scoring system when brain imaging is not available or ≥12 points when brain imaging is available) or definite (microbiologically confirmed *M tuberculosis*) tuberculous meningitis.^16^ We also used a composite microbiological reference standard of any positive CSF test (Ziehl–Neelsen stain microscopy, Xpert, Xpert Ultra, and MGIT) for *M tuberculosis*. Here, we included the index test (Xpert Ultra) in the reference standard as the existing tests are imperfect, and we judged the likelihood of false-positive detection of *M tuberculosis* DNA in the CSF of an HIV-positive person with aseptic meningitis in a tuberculosis-endemic area to be extremely low.

Baseline clinical characteristics were compared between those with definite tuberculous meningitis and the remaining participants, who were classified as other meningitis, via Wilcoxon rank-sum test for continuous variables and Pearson's χ^2^ squared or Fisher's exact test for categorical variables. We directly compared the sensitivity of Xpert Ultra with that of Xpert or MGIT using McNemar's test for paired categorical data. For the comparison of two diagnostic test performances against the primary reference standard, we used the Cochran–Mantel–Haenszel statistic for comparing matched categorical data across two strata of diagnostic tests versus the uniform case definition.^16^ Negative predictive value was calculated against the primary reference standard, both with and without the Xpert Ultra result being used to assign the uniform case definition category. Calculation of specificity and positive predictive value was done against the composite endpoint and the uniform case definition using 2 × 2 tables. We counted invalid tests (eg, culture contamination or Xpert error) as negative results. Additionally, a sensitivity analysis was done including only participants who received all three tests. We did univariate and multivariable logistic regression analysis to identify variables that correlate with microbiological confirmation of tuberculous meningitis. Variables which showed association in the univariate analysis (likelihood ratio test p<0·1) were eligible to be included in the multivariable model. Variables that were not normally distributed were log transformed.

Sample size calculation was done using the composite microbiological reference standard. For the paired analysis comparing the sensitivity of Xpert Ultra with that of Xpert or MGIT among participants positive for tuberculous meningitis, we assumed a third of pairs would give discordant results and that the sensitivity of Xpert Ultra would be 25% higher than that of Xpert and MGIT.^8^ Under these assumptions, we required 39 tuberculous meningitis cases to give 80% power with α set to 5%. Assuming the prevalence of tuberculous meningitis would be 20%, we aimed to recruit 200 participants.

We did a post-hoc analysis of the sensitivity of all assays according to the British Medical Research Council (MRC) tuberculous meningitis grade of disease severity grade against both reference standards (appendix p 12).

Stata version 13.1 was used for statistical analyses.

### Role of the funding source

The funders had no role in study design, data collection, data analysis, data interpretation, or writing of the report. The corresponding author had full access to all the data in the study and had final responsibility for the decision to submit for publication.

## Results

**Figure 1:**
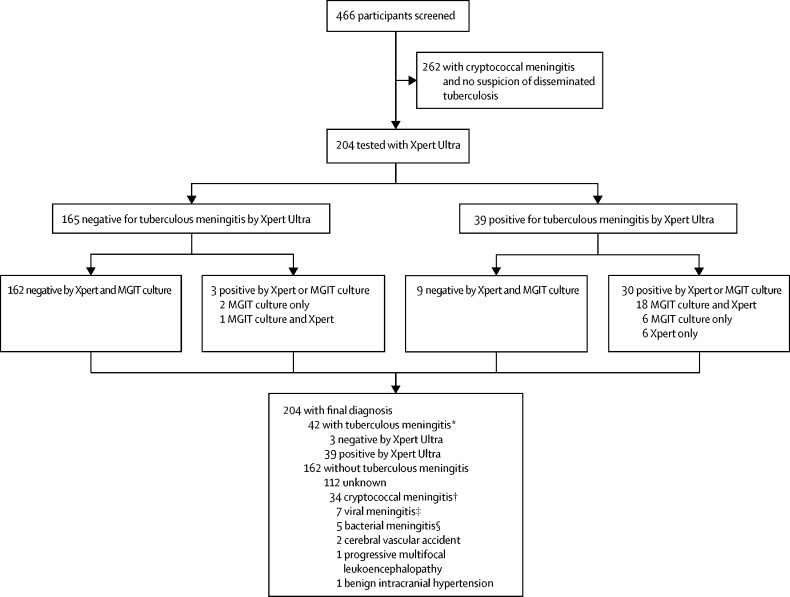
Flow diagram showing the diagnostic outcomes of the study population Xpert=Xpert MTB/RIF. MGIT=mycobacterial growth indicator tube. HSV=herpes simplex virus. CSF=cerebrospinal fluid. *Five participants with confirmed tuberculous meningitis had a positive CSF cryptococcal antigen test, of whom two had culture-confirmed cryptococcal meningitis. †Culture or PCR positive. ‡Two HSV type 1, two HSV type 2, two varicella zoster virus, and one cytomegalovirus. §One confirmed *Streptococcus pneumoniae* on PCR, four clinical diagnosis of bacterial meningitis based on CSF picture (ie, high CSF white blood cell count with neutrophil predominance suggestive of bacterial meningitis).

During the study period, 466 HIV-positive adults presented with suspected meningitis and consented to diagnostic lumbar puncture. Of these, 262 participants were diagnosed with cryptococcal meningitis and had no suspicion for disseminated tuberculosis warranting CSF tuberculosis diagnostics, so were excluded from this study. 204 were tested for tuberculous meningitis with Xpert Ultra (figure 1), of whom 195 (96%) were HIV positive with a median CD4 T cell count of 46 cells/μL (IQR 11–130). This number included 39 (19%) patients who had a positive CSF cryptococcal antigen test but were still tested for tuberculous meningitis because of suspected tuberculosis coinfection, and an additional 31 (15%) of those tested for tuberculous meningitis tested positive for cryptococcal antigen in serum but negative in CSF. Biofire meningoencephalitis PCR was done on 80 CSF samples; not all samples were tested owing to a technical issue with the machine. We collected a median of 11 mL (IQR 7–15) of CSF per participant, which left a median of 8 mL (5–11) of CSF to be spun down for tuberculosis testing after routine microbiology and chemistry testing had been done. Collected CSF was clear in 186 (91%) participants, with the remaining 18 (9%) having turbid CSF. Brain imaging was rarely done (ie, in 18 participants) because of a lack of functioning CT scanners in the study sites. No adverse events were attributable to the diagnostic testing (data not shown).

At baseline, lack of white blood cell pleocytosis and concentrations of glucose, protein, and lactate differed significantly between those with and without microbiologically confirmed tuberculous meningitis (table 1). The demographic, clinical, and diagnostic details of the 42 participants with microbiologically confirmed tuberculous meningitis are shown in the appendix (pp 5–6).

**Table 1:** Baseline characteristics of participants who underwent tuberculous meningitis testing

		**Definite tuberculous meningitis (n=42)**	**Other meningitis (n=162)**	**p value**
Age, years		32 (29–38)	35 (28–42)	0·43
Sex		..	..	0·73
	Female	19 (45%)	68 (42%)	..
	Male	23 (55%)	94 (58%)	..
HIV status		..	..	..
	Positive	41 (98%)	154 (95%)	0·47
	Negative	1 (2%)	6 (4%)	..
	Unknown	0	2 (1%)	..
On antiretroviral therapy		23 (55%)	99 (62%)	0·48
Headache duration, days		14 (7–21)	14 (5–21)	0·40
Glasgow Coma Scale		13 (10–14)	14 (13–15)	0·0012
CD4 count, cells per μL*		57 (13–108)	46 (10–188)	0·83
CSF opening pressure, cmH_2_O		20 (10–32)	18 (13–26)	0·91
Acellular CSF <5 cells per μL		11 (26%)	99 (61%)	<0·0001
CSF white blood cells per μL†		170 (70–283)	100 (40–275)	0·085
CSF lymphocytes		100% (84–100)	100% (80–100)	0·61
CSF total protein, g/L		1·2 (0·9–2·0)	0·3 (0·2–0·8)	<0·0001
CSF glucose, mmol/L		1·2 (0·9–2·0)	2·9 (1·9–4·4)	<0·0001
CSF lactate, mmol/mL‡		9·5 (4·6–11)	3·6 (2·4–5·1)	<0·0001
Alive at hospital discharge		25 (60%)	101/143 (71%)§	0·18

Values are n (%), n/N (%), or median (IQR), unless otherwise stated. p values are from Wilcoxon rank-sum for continuous data and Fisher's exact test for categorical data. Patients with definite tuberculous meningitis were positive for tuberculous meningitis by the composite microbiological reference standard. Patients with other meningitis were the remaining participants, including 112 with unknown causes and 50 with known causes of meningitis (see figure 1).

* 63 participants had CD4 count data (12 in the definite tuberculous meningitis group and 51 in the other meningitis group).

† Median values in participants with more than five white blood cells per μL of CSF.

‡ Lactate concentrations were available for 18 participants in the tuberculous meningitis definite group and 70 in the other meningitis group.

§ 19 participants had unknown status at discharge in the other meningitis group.

**Figure 2:**
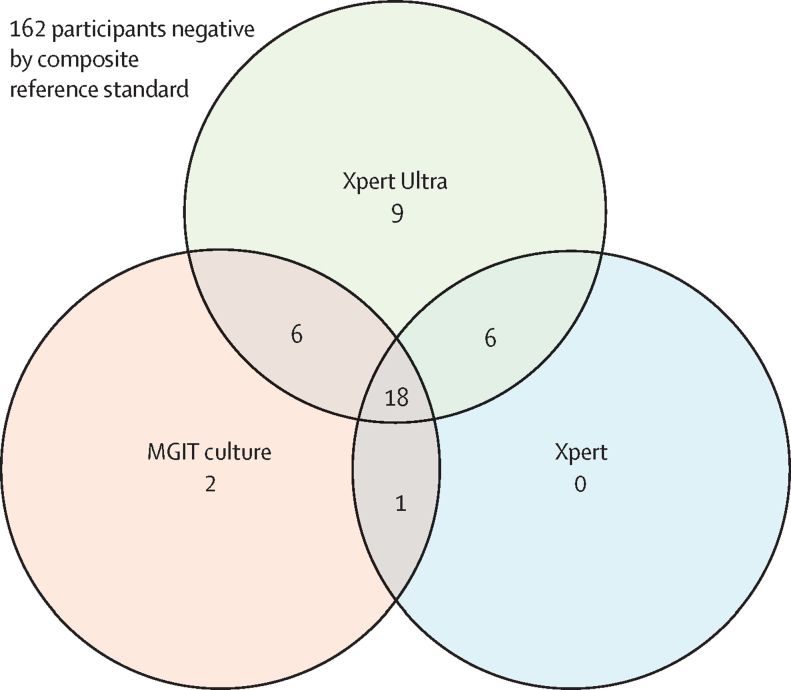
Venn diagram of positive diagnostic tests in the composite microbiological reference standard The Venn diagram displays 42 participants with microbiologically confirmed tuberculous meningitis by either Xpert, Xpert Ultra, or MGIT culture. Xpert=Xpert MTB/RIF. MGIT=mycobacterial growth indicator tube.

Of the 204 participants who underwent tuberculous meningitis diagnostic testing, Xpert Ultra was positive in 39, of whom Xpert was also positive in 24 and MGIT culture in 24 (figure 1). Xpert Ultra was negative in 165 participants, of whom 162 were negative and three were positive by the composite microbiological reference standard (figure 1). Xpert Ultra detected nine cases of tuberculous meningitis that were not identified by Xpert or MGIT culture (figure 2). We diagnosed probable or definite tuberculous meningitis in 51 (25%) of 204 participants when including the Xpert Ultra result in assigning the uniform case definition. When the Xpert Ultra result was excluded, 44 (22%) participants were classified as having probable or definite tuberculous meningitis. Of the 42 participants with definite tuberculous meningitis, five (12%) tested positive for cryptococcal antigen in CSF, including two (5%) who had positive fungal cultures (quantitative cryptococcal culture 520 000 and 20 000 CFUs per mL; figure 1).

We identified alternative causes of meningitis in 49 (30%) of the 162 participants without confirmed tuberculous meningitis (figure 1). In four patients without confirmed tuberculous meningitis who died in hospital, post-mortem examinations were done and causes of death were herpes simplex virus meningoencephalitis, progressive multifocal leukoencephalopathy, and disseminated tuberculosis without obvious CNS involvement in one patient; progressive multifocal leukoencephalopathy (PML) in one patient; pneumonia with no obvious CNS pathology in one patient; and meningoencephalitis with macroscopic appearance that was compatible with tuberculous meningitis, with histopathological confirmation awaited, in one patient.

Among the 42 participants with definite tuberculous meningitis, 17 (40%) died in hospital, with a median time to death of 4 days (IQR 2–6). Among the 162 patients in the other meningitis group, hospital outcome was known for 143, of whom 19 (13%) died in hospital.

When compared with the uniform case definition (probable or definite tuberculous meningitis), sensitivities were 76·5% (95% CI 62·5–87·2) for Xpert Ultra, 55·6% (44·0–70·4) for Xpert, and 61·4% (45·5–75·6) for MGIT (table 2). Against the composite microbiological reference standard, sensitivities were 92·9% (80·5–98·5) for Xpert Ultra, 65·8% (48·6–80·4) for Xpert, and 72·2% (55·9–86·2) for MGIT culture (table 2). Xpert Ultra was superior to Xpert in detecting tuberculous meningitis when either the composite microbiological reference standard or uniform case definition was used (table 2). Xpert Ultra was significantly more sensitive than MGIT culture when the uniform case definition was used but not when the composite microbiological reference standard was used (table 2). The negative predictive value of Xpert Ultra was 92·7% (87·6–96·2) against the uniform case definition (table 2). When including only the 117 participants who had received all three tests, no major difference in performance of any of the tests was observed (appendix p 7).

**Table 2:** Diagnostic performance of Xpert, Xpert Ultra, and MGIT culture for the diagnosis of tuberculous meningitis

	**Number**	Sensitivity *vs* composite microbiological reference standard*	**p value**†	**Sensitivity *vs* uniform case definition**	**p value**‡	**Negative predictive value *vs* uniform case definition**	**Specificity *vs* uniform case definition**
Xpert Ultra	204	92·9% (80·5–98·5); 39/42	..	76·5% (62·5–87·2); 39/51	..	92·7% (87·6–96·2); 153/165	100% (97·6–100); 153/153
Xpert	166	65·8% (48·6–80·4); 25/38	0·0063	55·6% (44·0–70·4); 25/45	0·0010	85·8% (78·9–91·1); 121/141	100% (97·0–100); 121/121
MGIT culture	142	72·2% (55·9–86·2); 27/37	0·092	61·4% (45·5–75·6); 27/44	0·020	85·2% (77·4–91·1); 98/115	100% (96·3–100);98/98

Values are percentage (95% CI); numerator/denominator unless stated otherwise. Xpert=Xpert MTB/RIF. MGIT=mycobacterial growth indicator tube.

* The composite microbiological reference standard included a positive CSF test on any of Ziehl–Neelsen stain microscopy, Xpert, Xpert Ultra, and MGIT culture. Specificity (and the positive predictive value) versus the composite endpoint is by definition 100% as the index test is included in the reference standard of definite tuberculous meningitis. If the Xpert Ultra result is excluded when assigning the case definition, the specificity of Xpert Ultra is 96% (95% CI 91–98; 153 of 160 patients) and the positive predictive value is 82% (66–93; 32 of 39).

† McNemar's test comparing the sensitivity of Xpert or MGIT culture with that of Xpert Ultra.

‡ Cochran–Mantel–Haenszel test comparing the distribution of Xpert or MGIT results with that of Xpert Ultra results against the uniform clinical standard of definite or probable tuberculous meningitis.

Of the 39 positive Xpert Ultra results, 14 were categorised as trace, ten as very low, seven as low, five as medium, and none as high; three results were unknown. Of the 14 Xpert Ultra trace samples, only four were positive on Xpert (three very low and one low) and six were positive on culture. Median time to CSF culture positivity was 14 days (IQR 10–15). Median time to positivity by Xpert Ultra semi-quantitative category was 15 days (13–15) for trace, 15 days (12–15) for very low, 12 days (9–21) for low, and 9 days (8–9) for medium (appendix pp 3, 8).

No cases of rifampicin resistance were identified with either Xpert or Xpert Ultra. Positive samples classed in the Xpert Ultra trace category have indeterminate rifampicin resistance because more than 16 CFUs per mL of *M tuberculosis* and amplification of *rpob* DNA is required to generate rifampicin susceptibility results;^13^ thus all 14 trace positive results had indeterminate rifampicin resistance in our study. Culture-based drug susceptibility testing was not done. MGIT culture reported contaminated results in 13 (9%) of 142 cultures performed.

When analysing by MRC grade, the sensitivity of Xpert Ultra against the composite microbiological reference standard was 100% in grade 1 disease, 96% in grade 2 disease, and 82% in grade 3 disease. Using the uniform case definition, the sensitivity of Xpert Ultra was 100% in grade 1 disease, 74% in grade 2 disease, and 69% in grade 3 disease. The observation of decreasing sensitivity with advancing disease MRC severity grade held true for Xpert and MGIT (appendix p 9).

The CSF from 45 participants with confirmed cryptococcal meningitis and no clinical suspicion of tuberculosis coinfection were all negative on Xpert Ultra, suggesting false positivity (eg, laboratory contamination) is a rare occurrence (specificity 100%, 95% CI 92–100).

Univariate analysis found that Glasgow Coma Scale score of less than 15, CSF pleocytosis, and lower CSF glucose, higher CSF protein, and higher CSF lactate concentrations were all positively associated with microbiological confirmation of tuberculous meningitis (table 3). Log_2_ CSF lactate concentration was not included in the multivariate analysis because of the large amount of missing data. In the multivariable logistic regression model, lower log_2_ CSF glucose concentration remained positively associated with microbiological confirmation of tuberculous meningitis, although the multivariable model analysis was restricted to 101 participants with a complete dataset for the variables included in the model (table 3). The median volume of CSF spun down for tuberculosis testing was 8 mL (IQR 5–11) and each doubling (ie, log_2_ increase) of CSF volume spun down increased the odds of tuberculosis confirmation by 40%, although this did not achieve statistical significance (table 3).

**Table 3:** Univariate and multivariable analyses of factors potentially associated with microbiological confirmation of tuberculous meningitis

		**Univariate model**			**Multivariable model**		
		n	Odds ratio (95% CI)	p value	n	Adjusted odds ratio (95% CI)	p value
Age, per year		204	0·98 (0·95–1·01)	0·26	..	..	..
Male sex		204	1·14 (0·58–2·26)	0·70	..	..	..
Duration of headache, per day		168	1·00 (0·98–1·01)	0·95	..	..	..
Glasgow Coma Scale score							
	15*	72	1 (ref)	..	35	1 (ref)	..
	11–14	97	3·62 (1·39–9·39)	0·0083	46	3·71 (0·84–16·48)	0·084
	≤10	33	5·50 (1·82–16·62)	0·0025	21	4·71 (0·93–23·82)	0·061
Log_2_ CD4 count, cells per μL		63	0·94 (0·71–1·34)	0·66	..	..	..
On antiretroviral therapy		202	1·34 (0·67–2·66)	0·40	..	..	..
Log_2_ CSF volume, mL		198	1·20 (0·84–1·72)	0·32	..	..	..
Log_2_ CSF volume spun down, mL		96	1·40 (0·84–2·35)	0·20	..	..	..
CSF pleocytosis†		189	4·94 (2·28–10·72)	<0·0001	101	2·04 (0·33–12·74)	0·44
Log_2_ CSF glucose, mmol/L		122	0·31 (0·18–0·54)	<0·0001	101	0·33 (0·17–0·65)	0·0010
Log_2_ CSF lactate, mmol/L‡		88	5·76 (2·45–13·52)	<0·0001	..	..	..
Log_2_ CSF protein, mg/dL		166	2·10 (1·52–2·89)	<0·0001	101	1·02 (0·56–1·87)	0·25

Variables that were significant (p<0·1) in the univariate model were included in the multivariable model. Odds ratios for log-transformed variables are per log_2_ increase. CSF=cerebrospinal fluid.

* p=0·0001 with the likelihood ratio test.

† >5 lymphocytes per μL of CSF.

‡ CSF lactate was excluded from the multivariable model because of the amount of missing data.

Of the nine participants who were positive only by Xpert Ultra, six had started antiretroviral therapy within the 6 weeks preceding the study (median duration 28 days, IQR 17–35), a timing consistent with unmasking immune reconstitution inflammatory syndrome.

## Discussion

In this HIV-positive population with suspected meningitis, Xpert Ultra showed higher sensitivity to detect tuberculous meningitis than either Xpert or MGIT culture against both considered reference standards, although the difference was not significant when Xpert Ultra was compared with MGIT culture against the composite microbiological reference standard. These findings from fresh CSF corroborate our earlier findings from cryopreserved CSF, supporting the robustness of these data.^8^

Importantly, more than a third (14 [36%] of 39) of positive Xpert Ultra results were in the trace category (correlating to <100 CFUs per mL), which is usually below the limit of detection of Xpert and potentially below the limit of detection of tuberculosis culture. Of these 14 trace positive samples, only four were positive by Xpert and six by culture. The ability to detect non-viable organisms is another reason why detection of tuberculosis might be improved with molecular tests versus culture.

As part of the uniform case definition, we did not include participants with possible tuberculous meningitis (n=74) in our analysis because of the non-specific nature of this category in this study population with advanced HIV—eg, due to concurrent neurological pathology such as PML (study population median CD4 T cell count 46 cells/μL, IQR 11–130).

With increasing market penetration of the Xpert platform in countries with high tuberculosis burden^24^ and a turnaround time of 84 min, Xpert Ultra is the best point-of-care test for tuberculous meningitis currently available and has been endorsed by WHO as the best initial test for tuberculous meningitis.^25^ Yet, our study shows a moderate 76·5% sensitivity of Xpert Ultra against a uniform case definition of probable or definite tuberculous meningitis, giving a negative predictive value of 92·7%. In other words, nearly a fifth of patients (nine [18%] of 51) with clinical phenotypes and CSF profiles highly suggestive of tuberculous meningitis and negative for all other tested pathogens were negative by Xpert Ultra (and by Xpert and culture). Four of these participants died from their illness, one with macroscopic post-mortem findings compatible with tuberculous meningitis. Our belief is that a proportion of these are false-negative results, but this remains to be determined in future studies. CSF could also be tested with Xpert Ultra on multiple days if safe to do so and a false-negative result is suspected; we have employed this strategy with success in several patients (outside of this study). Markers of host response should also be considered as potential future adjunctive diagnostic tests, although to date, none have been successful.^26^ Currently, clinical judgment remains relevant when making treatment decisions regarding tuberculous meningitis.^11^

Multivariable analysis showed low CSF glucose to be associated with microbiologically confirmed tuberculous meningitis. CSF lactate could not be taken through to the multivariable model because of the amount of missing data but was found to be strongly associated with microbiologically confirmed tuberculous meningitis in the univariate model and thus warrants further investigation as a diagnostic marker. Handheld glucometers and lactate meters are true point-of-care tests, with results available at the bedside within a matter of seconds. A low CSF glucose concentration (<2·2 mmol/L, <40 mg/dL, or CSF to plasma glucose ratio <50%) in a patient with symptoms and signs suggestive of tuberculous meningitis, once bacterial meningitis and cryptococcus have been excluded, is a good indication to consider antituberculous therapy regardless of tuberculosis-specific test results. The potential for using CSF glucose and lactate as components of a comprehensive diagnostic algorithm focused on probability of diagnosis should be explored in future studies.

We believe that using Xpert Ultra on CSF samples is highly specific for the diagnosis of tuberculous meningitis, although the absence of a perfect reference standard makes this difficult to prove. Furthermore, the inclusion of Xpert Ultra in the composite reference standard risked incorporation bias and is a limitation of this study. Yet, unlike sputum from the lungs, which can remain positive in the trace category on Xpert Ultra even years after treatment of prior pulmonary tuberculosis,^27^ CSF is a sterile body fluid that is replenished four to five times a day.^28^ Presence of *M tuberculosis* in the CSF progresses to death if untreated. In this study of hospitalised patients with advanced HIV who were from a tuberculosis-endemic country and had symptoms of subacute meningitis, we feel that the probability of a false-positive DNA-based test is negligible.

The median total volume of CSF spun down for tuberculosis testing was 8·0 mL; however, this volume was then divided into four, with a median volume of approximately 2·0 mL per diagnostic test. We used an algorithm for stepwise dropping of tests when the CSF volume collected was less than 6 mL so the minimal input CSF volume would always be at least 2·0 mL per diagnostic test to maximise the likelihood of obtaining an accurate result for the patient. Each doubling of CSF volume spun down increased the odds of tuberculosis confirmation by 40%, although this association was not significant. Xpert has been noted in some studies to have improved performance when higher CSF volumes are used; this study cannot inform whether the same is true for Xpert Ultra.^5^, ^6^ The stepwise use of tests on low-volume samples resulted in small samples receiving fewer tests and thus imperfect matching, with CSF from 204 patients tested with Xpert Ultra compared with only 166 with Xpert. There were also the common problems of culture, beyond diagnostic delay; only 142 culture results were received (including 13 that were contaminated) because of a number being lost when sent to an external mycobacterial laboratory.

11 (26%) of 42 patients with microbiologically confirmed tuberculous meningitis had an acellular CSF (fewer than five white blood cells per μL). All those with acellular CSF also had CSF opening pressure and total protein within the normal range and lower median CSF lactate concentrations (CSF lactate 4·6 mmo/mL *vs* 9·5 mmol/mL in the definite tuberculous meningitis group). We previously reported that one in three patients with definite tuberculous meningitis have acellular CSF, a finding that has also been noted in other HIV-positive cohorts outside of Asia.^29^ This observed absence of an inflammatory response in patients with microbiologically confirmed tuberculous meningitis could be explained by immune paresis secondary to advanced HIV disease (CD4 count was available in two of 11 patients and was three cells per μL and six cells per μL, respectively), or by early presentation (three of 11 patients had MRC grade 1 disease). Mortality was high in this subgroup despite the apparent absence of CSF inflammation, with six (55%) patients dying in hospital. Low CSF white blood cell count has been associated with death in a multivariate prognostic model in Vietnamese adults with tuberculous meningitis, and it is becoming increasingly clear that both absence of inflammation, as well as excessive inflammation, are deleterious in tuberculous meningitis.^2^, ^30^

The sensitivity of all assays was highest in early disease (MRC grade 1), and sensitivity decreased as disease severity progressed. Although the numbers are small and the analysis of sensitivity by MRC grade was post hoc, this finding supports the notion that an increasing immune response (ie, inflammation) aimed at controlling bacillary load is the mediator of disease. Thereby, an inverse relationship might actually exist between CSF bacillary load and disease severity in this population. This hypothesis warrants further investigation in immunology studies and highlights the importance of finding the optimal host-directed therapy to control damaging inflammation in this population.

When translating these results to the field, it is important to consider that in many hospital laboratory settings, centrifuging the CSF might not be feasible, in which case we would advise loading 2 mL of CSF directly into the Xpert Ultra cartridge to maximise the bacillary load, as dilution with sample reagent is not required for CSF. Additionally, although our cohort cannot inform diagnostic performance in an HIV-negative population, our paired analysis of specimens shows that Xpert Ultra performs better than Xpert in an HIV-positive population. Whether Xpert Ultra can improve outcomes of tuberculous meningitis or lessen unnecessary exposure to tuberculous meningitis medications remain to be determined. In this prospective study, with real-time Xpert Ultra results available to the clinical team within 24 h, in-hospital mortality was 40% (17 of 42 patients) compared with 50% (11 of 22) in our previous study on cryopreserved CSF in which results were not available to guide management decisions.^7^, ^8^ We cannot say whether this observed reduction in mortality is attributable to the availability of real-time Xpert Ultra results because this study was not designed to study the effect of Xpert Ultra on clinical outcomes. Additionally, this study included two clinical sites (Mulago and Mbarara hospitals), whereas the previous study was done solely in Mbarara hospital. Furthermore, given the time between this study and the previous study, additional potential confounding factors might be present that contribute towards the mortality difference. In addition to access to improved point-of-care diagnostics, earlier presentation to hospital, better supportive care, and optimised antimicrobial and anti-inflammatory treatments are required to reduce the high case-fatality in this population. Larger structural factors, such as poverty and weak health systems, also continue to play a role in the high mortality of tuberculosis and HIV coinfection and need to be addressed. At present, regular use of empirical therapy continues to be required for tuberculous meningitis.

In conclusion, Xpert Ultra offers a substantial improvement in rapid and accurate diagnosis of tuberculous meningitis, and is superior to Xpert. However, Xpert Ultra does not represent a perfect rule-out test. Clinical judgment and empirical therapy remain important to improve outcomes for patients with tuberculous meningitis. In this exciting era of rapidly evolving molecular diagnostics and biomarkers, the development of a highly sensitive point-of-care test that can facilitate rapid treatment and give clinicians confidence in rationalising the use of empirical tuberculosis treatment is a realistic goal.
