# Supplementary material for: Xpert MTB/RIF Ultra for the diagnosis of HIV-associated tuberculous meningitis: a prospective validation study
Source: Lancet Infect Dis. 2020 Mar;20(3):308–17. doi: 10.1016/S1473-3099(19)30550-X (PMC7045085; doi:10.1016/S1473-3099(19)30550-X)
Supplement: Supplementary appendix [file mmc1.pdf]

THE LANCET Infectious Diseases

Supplementary webappendix

This webappendix formed part of the original submission and has been peer reviewed. We post it as supplied by the authors.

Supplement to: Cresswell FV, Tugume L, Bahr NC, et al, on behalf of the ASTRO-CM team. Xpert MTB/RIF Ultra for the diagnosis of HIV-associated tuberculous meningitis: a prospective validation study. *Lancet Infect Dis* 2020; published online Jan 7. https://doi.org/10.1016/S1473-3099(19)30550-X.

Contents

Supplemental Figure 1. CSF flowchart illustrating diagnostic testing performed 2

Supplemental Figure 2. Days to MGIT culture positivity by Ultra semi-quantitative category 3

Supplemental Table 1. Step-wise testing of CSF in low volume CSF samples 4

Supplemental Table 2. Characteristics of participants with microbiologically confirmed TBM 5

Supplemental Table 3. Diagnostic accuracy when only participants whose CSF was tested with all 3 assays were included. 7

Supplemental Table 4. Ultra and Xpert semi-quantitative categories and days to culture positivity 8

Supplemental Table 5. Sensitivity of assays by MRC disease severity grade 9

Supplemental Figure 1. CSF flowchart illustrating diagnostic testing performed

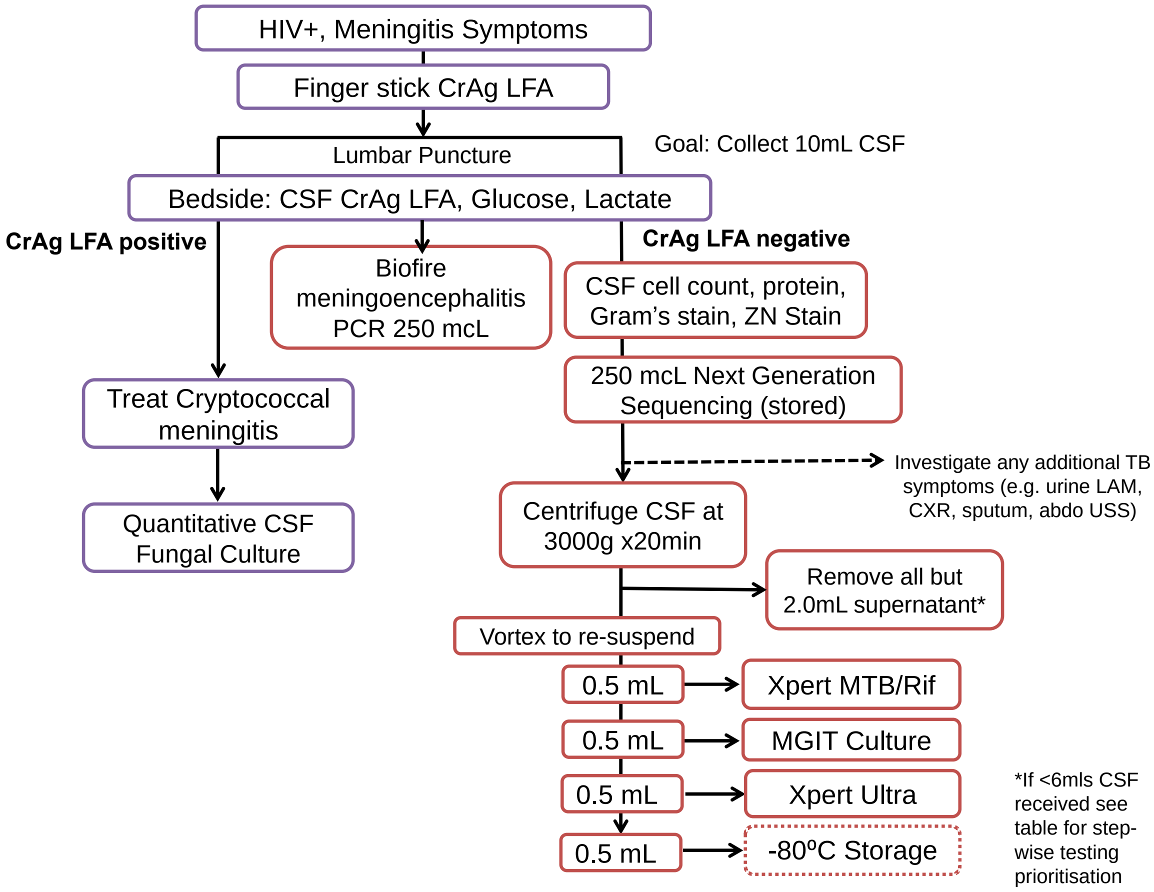

Biofire meningoencephalitis PCR was performed on 80/204 CSF samples. The machine had a technical fault for a period of time that precluded real-time testing on all participants.

Supplemental Figure 2. Days to MGIT culture positivity by Ultra semi-quantitative category

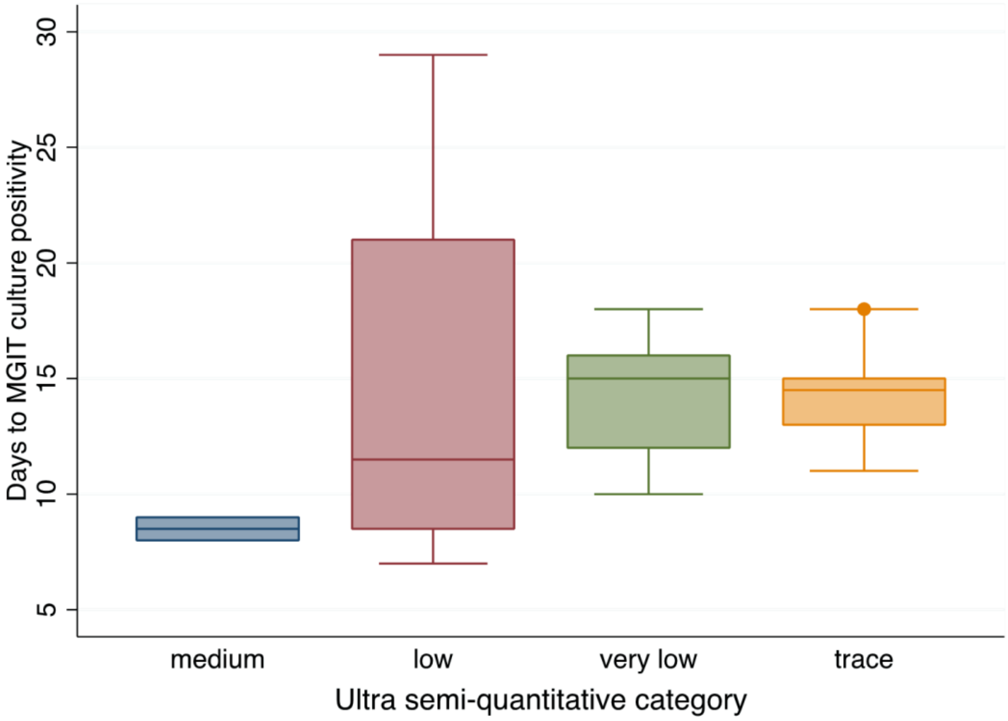

There were 36 participants with a known Ultra semi-quantitative category (5 medium, 7 low, 10 very low and 14 trace)

Supplemental Table 1. Step-wise testing of CSF in low volume CSF samples

Volume CSF received	WBC protein Gram	SPIN 3000g 20 minutes	Ultra (on 0.5ml pellet)	MGIT culture (on 0.5ml pellet)	Xpert (on 0.5ml pellet)	Cell pellet storage
<2mls	yes		yes	no	no	no
2-4mls	yes		yes	yes	no	no
4-6mls	yes		yes	yes	yes	no
>6mls	yes		yes	yes	yes	yes

Supplemental Table 2. Characteristics of participants with microbiologically confirmed TBM

Age (years)	Sex	CD4 (cells/µL)	ART (duration, days)	GCS	MRC grade	Duration headache (days)	Serum CrAg	CSF CrAg	CSF opening pressure (mm)	CSF WBC (cells/µl) [%lymphs]	CSF protein (g/L)	CSF glucose (mmol)	Ultra	Xpert	MGIT culture	Ultra semi-quant category	Days to MGIT culture positivity	Hospital outcome	Duration hospitalisation/time to death (days)
**35**	M	79	Yes (13)	12	2	30	+	+	150	115 [100%]	157	3·3	+	Neg	not done	trace		alive	13
**37**	F	461	No	14	2	14	Neg	Neg		unknown	320		+	+	+	very low		alive	9
**26**	M	19	Yes (35)	6	3	30	+	+	300	205	100		+	Neg	Neg	trace		died	17
**30**	M	-	Yes (11)	11	2	7	Neg	Neg	315	165 [100%]	154	1·1	+	+	Neg	trace		died	1
**37**	M	-	No	12	2	30	Neg	Neg		unknown			+	Neg	not done	low		alive	
**31**	M	-	No	13	2	14	Neg	Neg	90	<5	11	1·8	Neg	+	+	unknown	29	died	7
**52**	F	6	No	5	3	30	Neg	Neg	60	<5	21	1·8	+	+	Neg	very low		alive	
**32**	F	78	No	8	3	21	Neg	Neg	500	110 [100%]	105		Neg	Neg	+		14	alive	10
**32**	M	1	Yes (11)	9	3	21	+	+	520	40 [65%]	210	3·3	+	Neg	Neg	trace		died	2
**38**	M	36	Yes (69)	13	2	0	Neg	Neg		unknown		4·1	+	+	Neg	trace	unknown	alive	
**26**	M	93	Yes (3)	13	2	7	Neg	Neg		385 [80%]		1·2	+	Neg	+	very low		alive	1
**29**	M	-	Yes (675)	12	2	0	Neg	Neg	100	<5			+	+	+	unknown	11	died	5
**48**	M	27	Yes (228)	14	2	4	Neg	Neg		14 [60%]	54	3·4	+	Neg	+	unknown	14	died	6
**32**	M	3	Yes (5)	15	1	30	+	+	46	<5		6·7	+	+	+	very low	16	alive	22
**38**	F	170	Yes (1984)	12	2	7	Neg	Neg		20 [65%]			+	+	+	unknown	20	died	0
**48**	F	-	Yes (1310)	15	1	14	Neg	Neg		245 [100%]	119		+	+	+	low	13	alive	12
**34**	M	-	No	15	1	0	Neg	Neg		60	117	1·3	+	+	Neg	medium		alive	3
**38**	M	-	Yes (13)	13	2	30	Neg	Neg	230	75 [100%]	177	0·9	+	+	+	very low	15	died	5
**37**	M	-	No	10	3	14	Neg	Neg		65 [100%]	200	1·7	+	+	+	trace	13	alive	10
**36**	F	-	No	-		0	Neg	Neg		710 [100%]	250	1·8	+	+	+	medium	8	died	1
**34**	F	-	Yes (1568)	8	3	7	Neg	Neg		160 [100%]	211	0·6	+	+	+	trace	11	died	2
**32**	M	-	No	14	2	7	Neg	Neg	430	255 [100%]	184	0·7	+	+	+	very low	12	died	4

**29**	M	-	Yes (1824)	14	2	30	Neg	Neg	330	590 [100%]	419		+	+	+	medium	9	alive	10
**23**	F	-	Yes (93)	15	1	21	Neg	Neg	325	315 [100%]	72	0·9	+	+	+	very low	16	alive	5
**30**	M	-	No	15	1	0	+	Neg	45	<5	24	2·2	+	Neg	+	trace	15	alive	9
**44**	F	-	Yes (9)	5	3	0	Neg	Neg	140	<5	23	0·8	+	+	+	very low	12	alive	14
**32**	F	-	No	13	2	14	Neg	Neg	400	425 [100%]	217		+	+	+	low	10	died	4
**40**	M	-	Yes (40)	10	3	14	Neg	Neg	210	55 [100%]	119	1·6	+	Neg	Neg	trace		alive	8
**56**	F	-	Yes (176)	14	2	14	Neg	Neg	200	220 [100%]	199	0·7	+	+	Neg	medium		alive	12
**39**	F	-	No	14	2	21	Neg	Neg		65 [100%]	101	1·2	+	Neg	+	trace	18	alive	4
**23**	F	-	Yes (2675)	8	3	7	Neg	Neg	220	<5	49		Neg	Neg	+		2	died	29
**26**	M	-	No	14	2	14	Neg	Neg	290	200 [100%]	195	0·9	+	Neg	+	trace	14	alive	5
**48**	M	-	No	14	2	14	Neg	Neg	100	<5		1·1	+	Neg	+	trace	15	died	8
**28**	M	-	Yes (21)	14	2	21	Neg	Neg		175 [0%]	232		+	not done	not done	trace		alive	23
**32**	M	-	Yes (342)	14	2	14	Neg	Neg	165	<5	99		+	+	Neg	medium		alive	9
**29**	F	-	No	14	2	14	+	+		625 [83%]	187		+	+	+	very low	10	died	3
**28**	F	HIV neg	No	13	2	99	Neg	Neg	35	150 [63%]	116	2·6	+	not done	not done	low		alive	9
**41**	F	-	No	13	2	14	Neg	Neg	240	250 [90%]	107	0·9	+	+	+	very low	13	alive	13
**30**	M	-	Yes (95)	15	1	90	Neg	Neg	180	<5	24		+	+	+	low	7	died	7
**26**	F	-	No	9	3	0	Neg	Neg	15	165 [85%]	103	1·1	+	+	+	low	29	alive	14
**32**	F	-	No	7	3	14	Neg	Neg		<5	12	1·1	+	not done	not done	low		died	1
**29**	F	122	Yes (35)	14	2	24	Neg	Neg	200	310	184	1·1	+	not done	Neg	trace		alive	15

Supplemental Table 3. Diagnostic accuracy when only participants whose CSF was tested with all 3 assays were included.

CSF Test	N	Sensitivity vs. composite microbiologic endpoint [a]	Sensitivity vs. case definition [b]	Specificity vs. case definition	NPV vs. case definition
**Xpert Ultra**	117	91·4% (32/35) [76·9–98·2]	80·0% (32/40) [64·4–90·9]	100% (77/77) [95·3–100]	90·6% (77/85) [82·3–95·8]
**Xpert MTB/Rif**	117	68·6% (24/35) [50·7–83·1]	60·0% (24/40) [43·3–75·1]	100% (77/77) [95·3–100]	82·8% (77/93) [73·6–89·8]
**MGIT Culture**	117	74·3% (26/35) [56·7–87·5]	65·0% (26/40) [48·3–79·4]	100% (77/77) [95·3–100]	84·6% (77/91) [75·5–91·3]

[a] Composite microbiological endpoint included any positive CSF test including ZN stain microscopy, Xpert, Xpert Ultra, or MGIT culture.

[b] Composite case definition for probable or definite TBM is as per the published uniform definition

Supplemental Table 4. Ultra and Xpert semi-quantitative categories and days to culture positivity

Semiquantitative (SQ) category	Xpert (n=25)	Ultra (n=39)	Days to culture positivity by Ultra SQ category	
			n	Median days [IQR]
Unknown	2 (8%)	3 (8%)	··	··
High	0	0	0	··
Medium	0	5 (13%)	2	8·5 [8–9]
Low	13 (52%)	7 (18%)	4	11·5 [8.5–21]
Very low	10 (40%)	10 (26%)	9	15 [12–16]
Trace	NA	14 (36%)	6	14·5 [13–15]

Supplemental Table 5. Sensitivity of assays by MRC disease severity grade

MRC grade*	Ultra sensitivity		Xpert sensitivity		MGIT sensitivity	
	Composite	Uniform case definition	Composite	Uniform case definition	Composite	Uniform case definition
Grade 1 (n=67)	100% (6/6)	100% (6/6)	83% (5/6)	83% (5/6)	83% (5/6)	83% (5/6)
Grade 2 (n=100)	96% (23/24)	74% (23/31)	67% (14/21)	54% (14/26)	75% (15/20)	58% (15/26)
Grade 3 (n=33)	82% (9/11)	69% (9/13)	50% (5/10)	42% (5/12)	60% (6/10)	55% (6/11)

*MRC grade unknown in n=4

British MRC severity grade 1 = GCS 15 and no focal neurology; MRC grade 2 = GCS 11-14 with or without focal neurology or GCS 15 with focal neurology; MRC grade 3 = GCS$\leq$10 with or without focal neurology.
